# Imported malaria among African immigrants: is there still a relationship between developed countries and their ex-colonies?

**DOI:** 10.1186/1475-2875-8-111

**Published:** 2009-05-22

**Authors:** Juan Pablo Millet, Patricia Garcia de Olalla, Joaquim Gascón, Jordi Gómez i Prat, Begoña Treviño, M Jesús Pinazo, Juan Cabezos, José Muñoz, Francesc Zarzuela, Joan A Caylà

**Affiliations:** 1 Epidemiology Service, Public Health Agency of Barcelona, Pza Lesseps, 1, 08023 Barcelona, Spain; 2 CIBER de Epidemiología y Salud Publica (CIBERESP), Spain; 3 Hospital Clínic, International Health Center (CRESIB), IDIBAPS, University of Barcelona, Villarroel 170, 08036 Barcelona, Spain; 4 Tropical Medicine and International Health Unit, Primary Health Care Drassanes Center, Avda Drassanes 17-21, 08001 Barcelona, Spain

## Abstract

**Background:**

The objective of this study was to compare cases of imported malaria originating from the Spanish ex-colony of Equatorial Guinea (EG) with those originating from the rest of Africa (RA).

**Methods:**

All the African cases detected in Barcelona between 1989 and 2007 were investigated in a retrospective analysis. Clinical-epidemiological variables such as sex, age, *visiting friends and relatives *(VFR), species, hospital admission and chemo-prophylaxis were compared. Data were analysed by logistic regression, calculating the Odds Ratio (OR) and 95% Confidence Intervals (95% CI).

**Results:**

Of the 489 African patients, 279 (57,1%) had been born in EG and 210 (42,9%) in the rest of Africa. The cumulative incidence of imported malaria among those from EG was 179.6 per thousand inhabitants, while in those from the RA it was 33.7 per thousand (p < 0.001). Compliance with chemoprophylaxis (CP) was very low, but there were no differences between the two groups. Comparing those from EG to those from RA, the former were characterized by having more patients in the *visiting friends and relatives *(VFR) category, and more individuals younger than 15 years or older than 37 years, and more women. They also visited a traveller's health centre more often, had fewer hospital admissions and were less likely to reside in the inner city.

**Conclusion:**

Cases of imported malaria originating in Africa, are more likely to come from the Spanish ex-colony of EG, and VFR are more likely to be affected. It is recommended that developed countries promote prevention programmes, such as CP advice directed at African immigrants, and develop programmes of cooperation against malaria in their ex-colonies.

## Background

The population increase in endemic countries together with the increase in international travel and of migratory movements have made the worldwide risk of malaria higher now than it ever was [1,2]. There are an estimated 350 million cases worldwide annually, the majority of which occur in Africa [1], and this severely inhibits the economic development of many countries [3].

In developed countries, imported malaria predominates in tourists and in immigrants who travel to their country to *visit friends and relatives *(VFR). Despite measures to prevent malaria, this creates an appreciable morbidity and mortality [4-6]. As is the case in the USA [7,8] and other European countries [5,9,10], immigrants from sub-Saharan Africa account for most cases seen in Spain [11,12], and they are also those who are the least likely to take chemoprophylaxis (CP), and who are the most affected by *Plasmodium falciparum *[13-15]. In Barcelona, cases coming from African immigrants, particularly Equatorial Guinea (EG), a Spanish ex-colony where malaria is endemic [16], are the most common. However, few studies have described the characteristics and features of imported malaria among the most affected groups in various receiving countries with respect to other immigrant groups [17,18]. Moreover, although the importance of VFR as a factor associated with imported malaria has been emphasized in various studies [5,19], few of these studies have quantified this effect among the most affected groups of immigrants in the receiving cities.

The objective of this study was to characterize the group of immigrants that contributes most cases of imported malaria, specifically comparing those from EG, an ex-colony of Spain, and to cases originating from other African countries, in a cosmopolitan city such as Barcelona.

## Methods

### Population and period of study

The city of Barcelona, with an area of 100.4 square kilometers, has a population of 1,595,110 inhabitants, 15.6% from other countries (January 2007 census) [20]. African cases of laboratory-confirmed malaria included in the Malaria Register of the Barcelona Public Health Agency (BPHA) Epidemiology Service between January 1^st ^1989 and December 31^st ^2007 were studied in a retrospective analysis of prospectively collected data. Cases that were not laboratory-confirmed, those residing outside the city, those without a contact address in the city and cases of relapse from the same infection were excluded.

### Epidemiological Survey

Reporting of cases malaria to the public healthcare system is mandatory throughout Spain. In Barcelona, active epidemiological surveillance is carried out, in which hospital admissions and microbiology listings are reviewed. Almost all cases are visited in two specialized units, regularly contacted by a public health nurse. After notification, an epidemiological survey is carried out, in which socio-demographic (sex, age, country of birth, place of residence, declaring center, hospitalization, country of travel), diagnostic (species, technique), chemoprophylaxis and treatment variables are collected, as well as the dates of onset of symptoms, diagnosis, and hospital admission and registration. In addition, the following information was collected: the endemic geographic areas visited in the 30 days prior to the onset of symptoms, or the last country visited if the patient returned from travel more than 30 days previously (which would apply to infections by *Plasmodium vivax/Plasmodium ovale*), the reason for travel and in the case of being an immigrant, if the reason for travel was to visit family and friends (VFR), work or volunteer work, or, if they had just arrived in Spain (recently-arrived immigrant), if this was verified by the municipal census. Immigrant resident is defined as an immigrant that lives in the city. Information about completion of the chemoprophylaxis treatment, and the area visited according to the risk level for multidrug-resistant *P. falciparum *transmission, as defined by the WHO (Types I to IV: minimum to maximum level of recommendation for prevention), was also noted [21].

### Laboratory

The most relevant microbiological data were the type of species detected and the method used for diagnosis. Diagnostic criteria included the microscope observation of parasites and identification of species on a Giemsa-stained thick and thin blood films. When the parasite was detected only in the thick and morphological characteristics were indeterminate in the thin blood film they were considered to be *Plasmodium sp*.

### Statistical analysis

All cases of malaria imported by a patient born on the African continent were selected for the analysis. Cases were classified as resident immigrants or recently arrived in the city, both for EG and for the rest of Africa (RA). Recently arrived patients were compared to those resident in the city, and also those born in EG were compared to other African citizens. For the purpose of this analysis, the immigrants' children were also considered immigrants, even if they were born in Barcelona.

A descriptive analysis of the data was carried out. For the quantitative variables, the median and interquartile range (IR) was calculated for those that did not follow a normal distribution, and for the categorical variables the percentages were calculated. At the bivariate level the chi-test was used, and the corresponding non-parametric tests, where appropriate. The cumulative incidence rates per 1,000 inhabitants during the study period were calculated for patients from EG and for those from the rest of sub-Saharan Africa living in Barcelona using the average population in the city (January 2007 census) according to place of birth (1,264 inhabitants from EG and 4,131 from the RA endemic areas). At the multivariate level, logistic regression was used, comparing immigrants born in EG to those born in the RA. The 95% Confidence Interval (95% CI) was calculated and a significance level of 5% (p < 0.05) was accepted as statistically significant. Statistical analyses were performed using SPSS version 13.0 and EpiInfo version 6.

## Results

**Figure 1:**
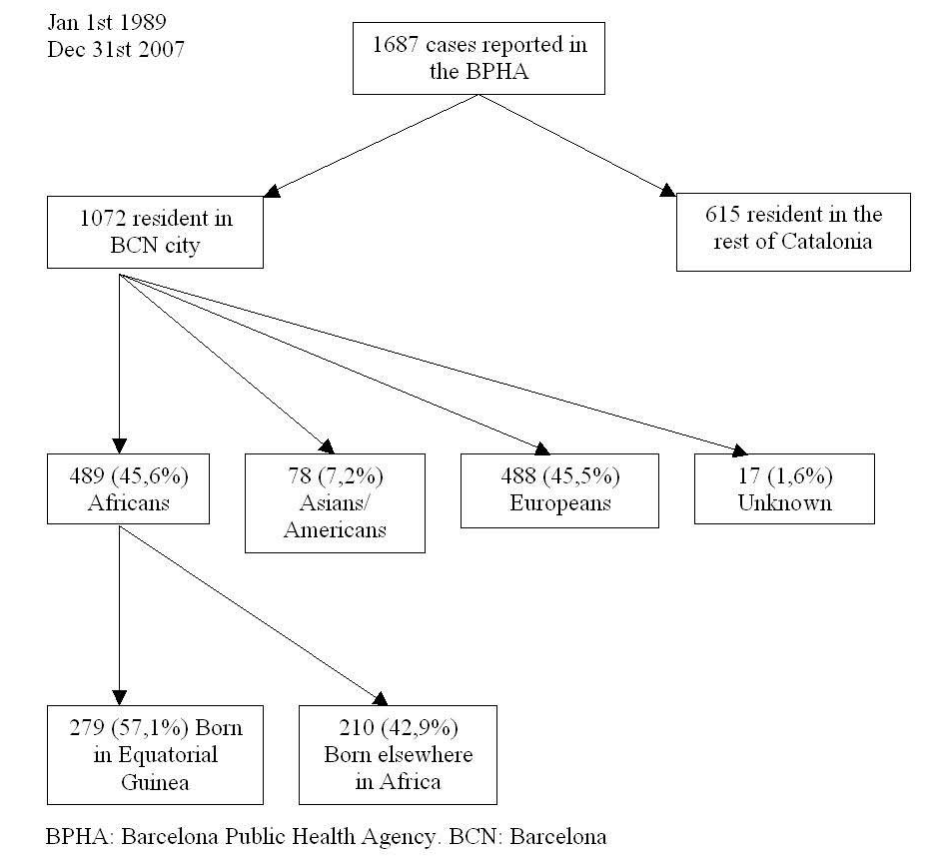
**Selection of patients with imported malaria according to place of birth**. Barcelona, 1989–2007.

**Figure 2:**
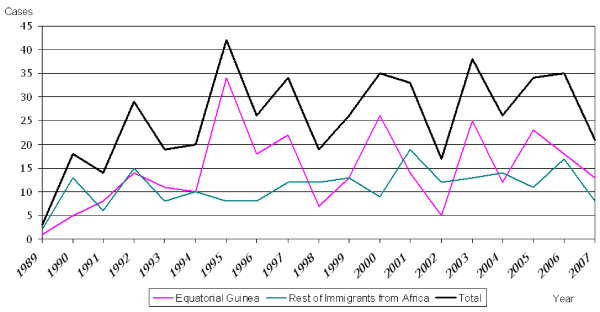
**Evolution of the recorded malaria cases**. Patients from Equatorial Guinea compared to other Africans. Barcelona, 1989–2007.

During the study period, a total of 1,663 cases of malaria were detected, 1,072 (64,5%) of which were residents in Barcelona. All of these cases were imported except two, one of which was induced by transfusion and the other was contracted from another affected individual through the sharing of venipuncture materials. Figure 1 shows the flow-chart for selection of patients according to place of birth. A total of 489 patients (45,6%) had been born in Africa, 57.1% (279) in GE and 42.9% (210) in the RA. Taking into account the country of birth, the cumulative incidence of imported malaria among sub-Saharan immigrants resident in the city was 179.6 per thousand inhabitants for the population of EG and 33.7 per thousand inhabitants for those from the RA, excluding the region of Maghreb (Morocco, Libya, Tunisia, Algeria and Occidental Sahara) (<0.001). Figure 2 shows the evolution of malaria cases among those born in EG and those born in the rest of Africa from 1989 to 2007.

Among the 489 Africans, 368 (75.3%) were residents in the city, 89 (18.2%) had recently arrived and in 32 cases (6.5%) it was not possible to determine if they were residents in Barcelona or not. Table 1 shows the different characteristics of the resident immigrants and of those recently arrived from Africa, who had been diagnosed with malaria in the city. Among the resident Africans the majority were VFR while the majority of those who had recently arrived had also traveled to Spain to visit friends and relatives (VFR, the reverse). Among the residents, 227 cases (61,7%) were from the population of EG and 141 (38,3%) from other African countries. Comparing the resident immigrants to those recently arrived, it was observed that the residents were predominantly from EG, VFR and infected by *Plasmodium sp*. (Table 1).

**Table 1:** Distinguishing characteristics between resident immigrants and recently arrived immigrants from Africa diagnosed with imported malaria in Barcelona city (1989–2007).

	Habitual Residents* 368/489 (75.3%)	Recently arrived 89/489 (18.2%)	ORc (95%CI)	ORa (95%CI)
Sex				
Male	178 (48.4)	45 (50.6)	1	-
Female	190 (51.6)	44 (49.4)	1.1 (0.7–1.8)	
Age				
15–38 years	228 (62)	58 (65.2)	1	-
< 15 years	65 (17.7)	13 (14.6)	1.3 (0.7–2.5)	
> 38 years	73 (19.8)	16 (18)	1.2 (0.6–2.1)	
Unknown	2 (0.5)	2 (2.2)	-	
Equatorial Guinea				
No	141 (38.3)	53 (50.6)	1	1
Yes	227 (61.7)	36 (40.4)	2.4 (1.5–3.9)	2.6 (1.6–4.3)
VFR				
No	9 (2.4)	12 (13.5)	1	1
Yes	359 (97.6)	49 (55.1)	6.9 (2.8–17.1)	6.8 (2.7–17.5)
Unknown	-	28 (31.5)	-	
Admission				
Yes	81 (22)	22 (24.7)	1	-
No	287 (78)	67 (75.3)	1.2 (0.7–2)	
Inner City Resident				
Yes	47 (12.8)	17 (19.1)	1	-
No	297 (80.7)	62 (69.7)	1.7 (0.9–3.2)	
Unknown	24 (6.5)	10 (11.2)	0.9 (0.4–2.2)	
Species				
Others	37 (10.1)	12 (13.5)	1	1
*P. falciparum*	282 (76.6)	72 (80.9)	1.3 (0.6–2.6)	1.7 (0.8–3.5)
*Plasmodium sp*	49 (13.3)	5 (5.6)	3.1 (1–9.8)	4 (1.2–13.6)
Declaring Center				
Others	64 (17.4)	59 (66.3)	1	-
HCP	58 (15.8)	17 (19.1)	0.7 (0.3–1.6)	
UMTSID	246 (66.8)	13 (14.6)	0.85 (0.4–1.6)	

* In 32 cases (6.5%) it was not know if the immigrants were habitual residents or recently arrived, so these were not analysed.

VFR: visiting friends and relatives, CV: residency in Inner city, HCP: Hospital Clinic, UMTSID: Unitat de Medicina Tropical i Salut Internacional Drassanes. ORc: crude *odds ratio*. ORa: adjusted *odds ratio*.

The median age of all of the 489 Africans was 29 years (IR 20–39), 27 years (IR 16–41) among those from EG and 30 years (24–37) for the RA (p = 0,089), with a slight predominance of women 249 (50.9%). A total of 68 patients (13.9%) lived in the inner city and 82 (16.8%) were children younger than 15 years. The most frequently isolated species was *P. falciparum *with 373 cases (76.3%), followed by *Plasmodium malariae *with 20 (4.1%), *P. vivax *with 19 cases (3.9%) and *P. ovale *with 14 (2.9%). In 63 cases (12.9%), the diagnosis was of *Plasmodium sp*. A total of 108 cases (22.1%) required hospital admission. In 359 cases (73.4%) the reason for travel was VFR. Among these, only a single case of 359 (0.3%) completed the CP, 18 (5%) did not complete it and 239 (66.6%) did not undergo CP. In 101 cases (28.1%), it was not known if CP had been carried out (Table 2). 99.6% of cases of imported malaria in Africans had visited a country with high risk of transmission of multidrug-resistant *P. falciparum *(Type IV Risk). The two cases (0.4%) that came from the region of Magreb (Morocco and Algeria) were *Plasmodium sp*. The median time period, from the commencement of symptoms until the diagnosis, was 6 days (IR 3–13), 5 days (2–14) for those from EG and 6 days (IR 4–10) for the RA (p = 0,23). During the period of the study no deaths among these immigrants were observed.

**Table 2:** Distinguishing characteristics of the African patients and factors associated with imported malaria according to source, Equatorial Guinea or the rest of Africa. Barcelona, 1989–2007.

	Equatorial Guinea 279 (57,1%)	Rest of Africa 210 (42,9%)	ORc (95%CI)	ORa (95%CI)	p-value
Age					
from 15 to 37 yr	153 (54.8)	153 (73.3)	1	1	-
Children < 15 yr	61 (21.9)	21 (10)	2.9 (1.7–5)	3.1 (1.7–5.8)	< 0.001
Older than 37 yr	62 (22.2)	33 (15.7)	1.9 (1.2–3.1)	2 (1.1–3.5)	0.02
Unknown	3 (1.1)	2 (1)	-	-	-
Sex					
Male	93 (33.3)	147 (70)	1	1	-
Female	186 (66.7)	63 (30)	5.1 (3.5–7.6)	4.8 (3.1–7.5)	< 0.001
Inner city Resident					
Yes	20 (7.2)	48 (22.9)	1	1	-
No	244 (87.5)	136 (64.8)	4.3 (2.5–7.6)	4 (2.1–7.6)	< 0.001
Unknown	15 (5.4)	26 (12.4)	1.4 (0.6–1.9)	1.1 (0.4–3)	0.79
Species					
Others	29 (10.4)	24 (11.4)	1	-	-
*P. falciparum*	213 (76.3)	160 (76.6)	1.1 (0.6–2)		
Plasmodium sp	37 (13.3)	26 (12.4)	1.2 (0.6–2.5)		
VFR					
No	42 (15.1)	56 (26.7)	1	1	-
Yes	221 (79.2)	138 (65.7)	2.1 (1.4–3.4)	2.2 (1.3–3.9)	0.004
Unknown	16 (5.7)	16 (7.6)	1.3 (0.6–3)	1.9 (0.7–4.8)	0.21
Hospital Admission					
Yes	39 (14)	69 (32.9)	1	1	-
No	240 (86)	141 (67.1)	3 (1.9–4.7)	2.2 (1.1–4.4)	0.02
Declaring Center					
HCP	24 (8.6)	58 (27.6)	1	1	-
UMTSID	214 (76.7)	112 (53.3)	4.6 (2.7–7.8)	3.2 (1.6–6.3)	0.001
Others	41 (14.7)	40 (19)	2.4 (1.3–4.7)	1.8 (0.8–3.8)	0.13
Chemo-prophylaxis*					
Correct	0 (0)	1 (0.7)	-	-	-
Not treated	149 (67.4)	90 (65.2)			
Abandoned	11 (5)	7 (5.1)			
Unknown	61 (27.6)	40 (29)			

M: median, RI: interquartile range, VFR: immigrant visiting friends and relatives, HCP: Hospital Clinic. UMTSID: Unitat de Medicina Tropical i Salut Internacional Drassanes. OR c: crude *odds ratio*. OR a: adjusted *odds ratio*.

* Calculations performed in a total of 359 VFR (indication of chemoprophylaxis).

Comparing the cases from EG to those from RA, at the bivariate level, the variables that were associated with the group from EG were age, sex, being VFR, residing outside the Inner City, visiting the Tropical Medicine and International Health Unit, Drassanes (UMTSID), and requiring fewer hospital admissions (Table 2). At the multivariate level, it was observed that imported malaria among the EG immigrants was more associated with women, children younger than 15 years, individuals older than 37 years and VFR immigrants, than among immigrants from the rest of Africa. Moreover, it was possible to observe that this group requires less hospitalization and visited the specialized center in the Inner City (UMTSID) more often, even though they live in less economically disadvantaged neighbourhoods (Table 2).

## Discussion

This study demonstrates that resident immigrants diagnosed with malaria in Barcelona are more frequently VFR, come from the Spanish ex-colony of EG, are infected by *Plasmodium sp*., and have some particular characteristics. Compared to other Africans, affected individuals from EG are predominantly women, children, persons older than 37 years and VFR immigrants. They are also less frequently admitted to hospital than other Africans and are less likely to live in the more disadvantaged districts.

The frequent observation of malaria in individuals of African origin is consistent with that observed in other European countries, like Great Britain and France [15,22,23], and the USA [24,25], which indicates a high prevalence of malaria in sub-Saharan countries. Despite the fact that few studies have characterized cases of imported malaria among African immigrants, in most countries and cities it can be expected that cases of malaria would be predominantly those imported by immigrants proceeding from ex-colonies or countries with a close post colonization relationship, as is the case in Spain with individuals from EG [12,26]. This is also probably true in Great Britain and France (Marseille), where many travelers and immigrants come from Kenya and Nigeria or Ghana and the Comoros Islands respectively [22,23,27], and also in the USA with refugee children from Liberia [28] and Ethiopian immigrants in Israel [29].

After comparing the incidence of imported malaria in the group from EG to those from the RA, the study shows that the disease is diagnosed more frequently among the EG patients. It may be that coming from an ex-colony, and therefore having generally lived longer in the receiving country, may carry particular advantages, such as a better understanding of the language, a greater facility for obtaining nationalization and better knowledge of the healthcare system. These individuals may have greater possibilities to travel under more legally and economically stable conditions. Despite having the same risk of becoming ill and the fact that preventative measures should be similar to other immigrants, the results could suggest the use of different intervention strategies at the community level. Moreover, this difference may also be due to the fact that health checks are occasionally carried out upon returning to one's own country and that these individuals probably receive visits from family members, who may occasionally avail of the visit to have a health-check carried out.

It was clear that the group of VFR immigrants carried the largest number of cases, perhaps because they travel more and, compared to the tourists, they take CP less often [12]. The low rate of compliance with CP is an extensive problem worldwide, and is likely to increase in cities with the increase in immigration proceeding from endemic regions, especially among VFR immigrants and their children [12]. Although studies carried out among healthy travelers have reported CP compliance of around 30%, such as in Paris [30], the low rate of CP observed in Barcelona imported malaria cases is similar to other cosmopolitan contexts such as New York, where none of the immigrants or their children had undergone CP [25], and Italy where the proportion of immigrants that had undergone CP was lower than 8% [31]. However, it is not possible to account for all of the VFR travelers who did not become ill because of correct CP treatment, or to quantify the importance of CP in preventing the disease. In recent years, UMTSID has performed malaria screening on some immigrants arriving from endemic countries. Another limitation of the study was the inability to differentiate cases in UMTSID with malaria diagnosed by screening from the symptomatic cases, or to distinguish the children of immigrants with acquired semi-immunity from those who were not semi-immune because of having been born and having lived in a non-endemic country.

The distinguishing features observed in the EG patients with imported malaria are likely to reflect, not so much the characteristics of the country of origin but rather of the greater number of years settled in the country, given it's relationship as an ex-colony. Established African immigrants in Barcelona are predominantly women, as is perhaps the case in other cities and countries [20]. Having higher risk of imported malaria could perhaps be because these individuals have traveled more and may be more concerned with their health and that of their children, rather than by virtue of being more susceptible to the disease.

The fact that none of the six deaths reportedly due to *P. falciparum *in Barcelona city during 1989–2007 occurred among immigrants (all were tourists [12]) and that the number of admissions and episodes of severe malaria among these individuals is low [12,32] suggests the persistence of a certain level of immunity among immigrants, despite having lived outside the endemic areas for a number of years [32,33]. This possibility is also supported by the high percentage of registered *Plasmodium sp*. The low level of parasitaemia observed among the immigrants made the identification of the species in the area more difficult. However, to assume that these patients have lower risk is not prudent [34,35], because they usually visit more rural areas and spend more time in zones without access to health services. Moreover, a particularly vulnerable group to consider, in addition to the tourists, would be the young children of the immigrants [12,28] born in Spain and therefore without acquired semi-immunity [25,35,36].

With regard to admissions, if the same level of natural acquired semi-immunity among sub-Saharan immigrants because of coming from hyper-endemic countries is assumed, the Guineans are probably admitted less often because the disease is detected earlier as these individuals have spent more time in Spain, and are more familiar with the language and the functioning of the health system. Also, the low number of hospital admissions among the EG population despite the high percentage of cases with *P. falciparum*, might be explained by the fact that many cases are detected by screening during the visit to the UMTSID [12] after returning from their country, despite being asymptomatic [26]. Moreover, it is important to note that there are fewer recently arrived immigrants, despite the possibility of civil registration and the advantages of access to a free health system, even for individuals who do not habitually live in the city.

In order to control malaria, it is essential to combine different strategies against both the parasite and also the vector to reduce human-vector contact [4,9,19,37-41]. To reduce the number of imported cases in the African VFR, specifically among those born in EG and their children, it is very important to improve prevention and promote CP through directed campaigns, to make medicines and the health system more accessible, and to inform about the symptoms of malaria, especially among the children of resident immigrants [15,27,35,36,42]. Promoting health education in the population, training qualified personnel, developing specific control programmes against the disease, and conducting research, both basic and epidemiological, are also key to fighting imported malaria. For this reason, it is very important that large cosmopolitan cities incorporate, as is being done in Barcelona, travelers health centers and centers of tropical medicine and international health, especially in the districts with highest immigration, and that protocols are established with the Primary Health Care system [43]. In cases with considerable cultural or language barriers the Community Health Workers could contribute to a better follow-up of the preventive measures and adherence to CP regime [5,15]. All of this would undoubtedly improve one of the most important aspects of the problem, the low perception of disease risk among African immigrants.

To make the various preventative measures mentioned more effective, it is important that developed countries identify the most abundant immigrant groups, and those with the highest probability of importing malaria. In the context of the increase in international travel and of migratory movements, it is recommended that countries encourage specific prevention programmes for CP treatment and other preventative measures among all immigrants, which could potentially be VFR and their children.

## Competing interests

The authors declare that they have no competing interests.

## Authors' contributions

JPM, PG and JC designed the study, collected the data, analyzed and prepared the first draft. All authors put forward different ideas, contributed to the interpretation of the data, early drafts and agreed the final draft.
